# Six lessons from first year COVID-19 restrictions: what can we do better in the future?

**DOI:** 10.1186/s12544-021-00513-2

**Published:** 2021-09-10

**Authors:** Yusak Susilo, Jonas Floden, Karst Geurs

**Affiliations:** 1 University of Natural Resources and Life Sciences, Vienna, Austria; 2 University of Gothenburg, Gothenburg, Sweden; 3 University of Twente, Enschede, The Netherlands

When the World Health Organization (WHO) declared the SARS-CoV-2 (COVID-19) outbreak a pandemic on March 11th 2020, authorities around the globe were urged to implement non- pharmacological interventions (NPIs) to decelerate infection rates (European Centre for Disease Prevention and Control, 2020). This led to many European countries to implement various measures to reduce the spread of the virus, ranging from banning unnecessary travel, to social distancing, to the closure of schools, restaurants, hotels, shops and other amenities, to the closing of borders. These measures have immediate impacts on the movement of both people and freight. From the reductions, re-arranging, rescheduling and cancelation of trips (e.g. [15, 20, 21, 29]) to shifting the activities into different destinations (e.g. [28]) or to the virtual realm (e.g. [3, 7, 23]), the measures have lead to significant impacts. An unprecedented disruptions of supply chains has been seen, causing impacts on food systems, public health, and employments.

Whilst there have been a lot of studies on the impacts of COVID-19 restrictions on transport and mobility, there have not been much a reflection on how we can do better if another pandemic strike in the future, and this is the objective of this editorial article. Based on the evidences drawn upon 11 papers from select European countries, which investigated wide range issues, from the readiness of port-state control inspection procedure [1], the impacts of COVID-19 restrictions to the work and travel patterns of civil servants in Sweden [14], the impacts of inter personal distance towards the sustainability operation of public transport in Italy [12], the willingness to pay to use the public transport and shared services given the COVID-19 circumstances in Spain [5], and the impacts of COVID-19 restrictions to travel and spatial movement patterns and trade-off behaviours of urban and rural residents in Germany [4, 16–18], Greece [26], Italy, India and Sweden [2, 8], there are common learned lessons that can serve as a guidelines on how we can deal with the disruptions better in the future.

In term of context, these case studies give us range of evidences from different settings such as Greece, which undertook strong measures to control COVID-19 pandemic while the number of cases was still low despite its difficult social and economic situations, to Sweden, a relatively wealthy Nordic country with a strong health care system, which implemented a relatively more relax measurements towards COVID-19.

## Lesson 1: Mobility impacts of COVID restrictions vary greatly across countries, geographic areas and demographic groups

Whilst it is clear from all the gathered evidences from all studied countries that there have been a significant reductions of movement due to the COVID-19 mobility limitation measures, the impacts are different across different socio-demographic groups, travel modes, trip purposes, and geographical locations. On average, there have been reductions of trip frequencies and public transport ridership has been decreased around 40–80%, an increase in cycling share, and, in some areas, also an increase in walking trips, in particular for health purposes. During the lockdown period in Spain, mobility to workplace dropped by 80%, compared to pre-COVID-19 trends [5]. The changes, however, less apparent in rural areas [4, 17] and also can be seasonal, in particular, due to the start and stop of school season [2]. In Germany, Kolarova et al. [16] found that travellers during the lockdown period switched to unimodal travel mode, e.g. the use of private car, and travel less often for daily needs. Bin et al. [8] did not find a strong correlation between the countries' restriction policy and the respondents' likelihood to adopt the new and online-based behaviours for any of the activities after the restriction period.

Many evidences also show that much of the reductions are also due to issues beyond transport and mobility. For example, the evidences show that only a particular type of workers can smoothly move to work-for-home and reduce their physical commute, whilst for some that is not the case. In Greece, for example, the reductions of mobility are also due to temporary unemployment of the work force (Helenic Statistical Authority 2020, as quoted by [26]).

Perception of safety and security were also consistently found significant in influencing people in choosing the destinations and time of their travel [8, 18]. For example, to avoid too much interactions with strangers, many elderly consciously avoid congestion in shops and services and only do their activities on off-peak periods. That is said, other than shopping during off-peak hours, on average, the elderly people seemed not to have reduced their daily trips to a large extent despite being encouraged to do so by the government and respective policy measures [26].

Furthermore the impacts of the measurements towards Covid19 are also different across household members. Male, in particular the ones of productive ages (40–60 s) and/or employed are the ones who still are likely to travel, 30–40% more than others, whilst elderly and women are the ones who have been reducing their travel to a greater extent [8, 17]. The reasons why women are more negatively impacted by COVID-19 restrictions may not only be because they are taking a greater responsibility for the inner household chores, such as taking care of children when schools are forced to close, but also that the type of industries that has a high share of female workers, e.g. service and hospitality industries, have been impacted by the pandemic to a greater extent [26].

At the same time, there are also long commuters who see the COVID-19 travel restrictions as a blessing in disguise because they can spend more time with their family (e.g. [17]). Furthermore, a study among a number of civil servant agencies in Sweden shows that women and older civil servants are happier with teleworking than their fellow civil servants [14]. Kalorova et al. [16] found that the typical people who shop online during the lockdown period were young females living in urban areas with previous experiences with online shopping and that assessed their risk of Coronavirus infection as high. Similar trends also found by Bin et al. [8].

Built environment and geographical location also matters. For example, Politis et al. [26] found that in the smaller cities (less than 100,000 people) the impact discrepancies among gender are more apparent. Evidence shows a higher proportion of reduction in travel among women in smaller cities, than among men. The evidence also show that in rural areas there are higher proportion of people that kept their travel patterns, as the spatial characteristics of rural areas may not allow them to travel and engage in activities differently [4, 17].

Overall, the fact that different demographic groups reacted differently during the lockdown period calls into question the assumption that population can be treated as homogeneous. It is also clear that measurements that may be suitable for urban settings cannot be simply transferred to rural areas. Future policies and measures should take into account this heterogeneity and act accordingly.

## Lesson 2: The local situation and individually perceived control matter

The effectiveness and suitability of COVID-19 measurements also depend on the local impact of the pandemic, norms and available opportunities. For example, based on a qualitative studies on a rural community in Germany, König and Dreßlerm [17] found that some respondents did not change their behaviour as they neither see significant changes in their immediate surrounding caused by the pandemic, nor do they perceive there is anything that they could do in dealing with the virus. Similar finding also found by Anke et al. [4].

There is a clear relationship between people perception of no need for changes or even resistance to change and non-compliance. Given the circumstance, König and Dreßlerm [17] argue the importance to introduce and promote the concept of Responsible Transport (originally promoted by Budd and Ison [9]) in recognition of the pandemic situation and for post-COVID recovery. Responsible Transport requires an element of individual responsibility that involves the decision whether traveling is really necessary or can be avoided and the impact of travel choices on others.

## Lessons 3: Evidence on long run mobility impacts is mixed

The evidence on the long term impacts of movement restrictions on the speed up of the digital transformation both in working and social life of people is so far mixed. Many respondents from different studies consistently indicated that they are not likely to continue working on-line to the same extent in the future as they have done during the pandemic [8, 17]. Anke et al. [4] argue that, whilst at this stage it is unclear if any of the reported short-term adaptation behaviours will translate into more permanent changes, it is also too early to say otherwise. Among Sweden public agencies’ employees, Hiselius and Arnfalk [14] found that whilst more than 90% of respondents believe themselves and colleagues has become much better at collaborating virtually, however, only 5% believe in a massive change in how they and their colleagues will work in the future. Hiselius and Arnfalk [14] concluded that, for case study, excluding office presence entirely does not seem to be a viable option, due to the risk of missing out on essential communication as well as social interaction. Bin et al. [8] found that the acceptance and long-term adoption of the online alternatives for activities are correlated with the respondents' personality and socio-demographic group, highlighting the importance of promoting alternatives as a part of longer-term behavioural and lifestyle changes.

Whilst the propensity of individuals to keep their on-line working and social engagement beyond the pandemic period is relatively small, the findings also consistently show that there are an agreement that people would travel, in particular flying, less [17] and may be cycle more in the future. Some government responses during the pandemic might have long standing mobility impacts. In many cities in Europe and North America, cycling infrastructure has been expanded and improved and traffic calming and vehicle restrictions have been implemented, or plans have been made to accommodate and encourage cycling (see [10], for an overview). Cycling increased considerably from 2019 to 2020 in most cities of Europe, North America, and Australia, and Buehler and Pucher argue this trend is likely to persist in over the coming years. That is said, whether such positive expectation would become reality remains to be seen. Some recent survey reports (e.g. [20]) indicate otherwise.

Furthemore, some behavioural change, e.g. digitalisation, may only long lasting if we can find the right balance between its positive and negative impacts. Given that lockdown has been facilitating a large scale introduction of digitalisation of all areas in one’s daily life, Kolarova et al. [16] suggest a possible mid- and long-term changes of working activities and travel in the future. At the same time, the evidences (e.g. [23–25]) also suggest that whilst working at home can improve well-being for some, it can also cause increase stress, exhaustion, anxiety, depressions and musculoskeletal issues, which can lead to much complex, negative, household dynamics [22]. Those struggling to be productive working at home often cited the need to provide care for family members or homeschooling children as a key barrier to productivity [13]. Thus, whilst indeed the transition towards digitalisation has been speeded up by the events during the pandemic that has forced people to try and learn new ways of working and in particular getting accustomed to the opportunities brought by digitalisation, whether such positive behavioural change can be retained depends on our ability to create supporting policies and environments that mitigate the negative impacts of such digitalised travel and activities.

## Lessons 4: Increased costs and reduced revenues challenges public transportation

One of transport sectors which suffers a lot due to COVID-19 is public transport. Some of evidences gathered by our papers show negative impacts to the people attitudes towards public transport. To keep people to use public transport during the pandemic period, there is a strong expectation among users for the operators to sanitise the vehicles for them. Based on 984 sample from Spain, Awad-Nunez et al. [5] found that measures such as the increase of supply and vehicle disinfection, result in a greater willingness to use public transport in post-COVID-19 times. Similarly, the provision of covers for handlebars and steering wheels also significantly increases individuals' willingness to use sharing services. However, respondents expect that these measures and improvements would be implemented while maintaining the same pre-COVID-19 ticket prices. This raise a question how the additional cost for such actions can be covered while still suffering from the reduction in passengers. In other words, the economic viability of such measures is something that need to further discussed and considered. Tirachini and Cats [27] noted that the decline of public transport use during the COVID-19 will lead into a financial crisis for public transportation operators. Just from the capacity reduction due to COVID-19 related measurement, Coppola and de Fabiis [12] estimate the High-Speed-Rail’s revenue in Italy declined about 20–60%. Kolarova et al. [16] argued that given the changed preferences towards the public transport during the lockdown period, short-term actions aimed at lowering the perceived infection risk by the users is necessary. It is important to remember that public transport demand might not return to pre-pandemic levels and bankruptcy of public transport providers is a possibility, if there is no intervention or support mechanism in place. Government decisions and the final duration of the COVID-19 crises is likely to determine if public transport supply and demand are affected in the next years after the end of the pandemic. In addition, long-term transport policy strategies and measures that aim to promote the use of public transport usage might have to be developed and implemented.

## Lesson 5: The importance of agile public transport operations under a supportive governance framework

The challenge for public transport operators is how to provide a high frequency, reliable and comfortable public transport service whilst keep the speed of contagion down and in an affordable manner. From demand perspectives, Almlöf et al. [2] indicate that during the pandemic the demand for public transport varies substantially as people from different socio-demographic groups responded differently towards the COVID-19 restrictions. This open an opportunity for the operators to reassign capacity to (lower income) neighbourhoods whom residents are more likely to still need to travel despite the COVID-19 restrictions due to the nature of their employments. Almlöf et al. [2] also argue the importance of authorities to also consider directing information regarding how to behave in public transport towards those more likely to use public transport, e.g. those with lower income or education level.

From supply perspective, some of COVID-19 related regulations that need to be implemented in public transport do provide some unexpected challenges. Using a high-speed rail case, Coppola and de Fabiis [12] show that one-meter interpersonal distancing on-board vehicles can be sustainable only with drastic limitations to travels and activities among the population. To cope with the current demand, an increase of line capacity up to 70%–80% is needed, whilst increasing frequency on peak time is not feasible and higher number of coaches per train could result in a train length greater than the length of the station platforms, which is also currently unfeasible. To cope with this, an advance travel information system, which helps spreading the demand across different time of day, and a seat booking system may ease the problem to some extent.

In encouraging people to work from home, Politis et al. [26] argue of the importance of employment and financial strategies that would help workers, in particular from low income group, to gradually transition to digital world. Targeted policy intervention is also required to support women during the pandemic, in particular on the one which contributes to the preservation of women’s employment opportunities.

Comparing the lockdown impacts between the German states with and without lockdown measures, Anke et al. [4] found that differences were only found in urban areas, and not in rural areas. König and Dreßlerm [17] argue that reduction of trips is not always as possible in rural areas as in urban areas where jobs that allow for remote working are more common. Thus, when having a possible next pandemic situation in mind, policy is challenged to ensure digital connections for facilitating remote working in rural areas.

In term of regular maritime inspection and control, Akyurek and Bolat [1] found that port state control inspection and ‘new inspection regime’ worked efficiently under the pandemic outbreak. However, since all inspections are carried out by the in-person presence, the human factor may be a treat for possible infection either for the ship crew and the Port State Control Officer. Thus, technological development on inspection methods such as remote inspections by media devices, should be considered.

## Lesson 6: Time to revisiting the thinking underlying our planning approach?

By end of the day, ‘accessibility’, not ‘mobility’—to people, goods, services e.g. shops, education, and to work (and income), healthcare, recreation—should be the key purpose of the urban transport system under the pandemic era [26]. More specifically, addressing the psychological consequences of fear, confinement and forced cohabitation or loneliness are strongly associated with the ability of people to have access to various activities with the use of the transport system [19].

Measures and policies after the pandemic should also give more emphasis on special user groups and especially to people with special mobility needs, elderly and other vulnerable citizens such as homeless and mobility-impaired who often feel that they are at the margin of our today’s societies. A particular challenge is that the needs in these groups may vary, especially under exceptional circumstances such as a pandemic.

## Conclusion: Importance of a deeper analysis on the revealed changes

The diversities in findings of these 11 papers demonstrate the immense complexity of situational and behavioural adjustment processes due the pandemic related measurements. It is paramount for us to not only focus on easing the negative impact of the pandemic but also to learn from the observed behaviours to date. Like it or not, many studies (e.g. [6, 11]) have argue that globalisation and climate change will make the occurrence of new diseases more common in the future. Thus, a robust, evidence-driven, possible solutions in dealing with a wide spread pandemic is desperately needed. As noted by König and Dreßlerm [17], whilst in the last one year there have been a significant accumulation of quantitative evidences based on secondary movement and traffic data, there are not much studies focuses on the decision making processes, psychological understanding and behavioural mechanism underlying such changes. This create gaps of our ability in dealing with equity, disparity and social justice issues in particular due to the direct and indirect impacts of the measures to reduce personal mobility, economic activities, and isolation, and is something that are needed to be addressed in the future.
